# Supplementary material for: Unveiling the Bioactive Potential of the Invasive Jellyfish Phyllorhiza punctata Through Integrative Transcriptomic and Proteomic Analyses
Source: Biomolecules. 2025 Aug 4;15(8):1121. doi: 10.3390/biom15081121 (PMC12383608; doi:10.3390/biom15081121)
Supplement: Supplementary file 1 [file biomolecules-15-01121-s001.zip › Suplementary Material/FileS3/PEPFOLD4/XP_065051153_13749/clusters.html]

 PEP-FOLD clusters


Download archive of all models  here   

|  |  |  |
| --- | --- | --- |
| # | file | sOPEP |
| # cluster 1 - Size: 15 |  |  |
|  | XP\_065051153\_13749-model1 | -30.6436 |
|  | XP\_065051153\_13749-model1.1 | -30.4715 |
|  | XP\_065051153\_13749-model1.2 | -30.305 |
|  | XP\_065051153\_13749-model1.3 | -30.2682 |
|  | XP\_065051153\_13749-model1.4 | -30.2326 |
|  | XP\_065051153\_13749-model1.5 | -30.1649 |
|  | XP\_065051153\_13749-model1.6 | -29.2638 |
|  | XP\_065051153\_13749-model1.7 | -29.1271 |
|  | XP\_065051153\_13749-model1.8 | -28.7844 |
|  | XP\_065051153\_13749-model1.9 | -28.7446 |
|  | XP\_065051153\_13749-model1.10 | -28.4348 |
|  | XP\_065051153\_13749-model1.11 | -28.3909 |
|  | XP\_065051153\_13749-model1.12 | -28.2113 |
|  | XP\_065051153\_13749-model1.13 | -26.7905 |
|  | XP\_065051153\_13749-model1.14 | -19.2649 |
| # cluster 2 - Size: 12 |  |  |
|  | XP\_065051153\_13749-model2 | -30.5655 |
|  | XP\_065051153\_13749-model2.1 | -30.1076 |
|  | XP\_065051153\_13749-model2.2 | -29.3073 |
|  | XP\_065051153\_13749-model2.3 | -28.9081 |
|  | XP\_065051153\_13749-model2.4 | -28.6077 |
|  | XP\_065051153\_13749-model2.5 | -28.4933 |
|  | XP\_065051153\_13749-model2.6 | -28.3377 |
|  | XP\_065051153\_13749-model2.7 | -28.2986 |
|  | XP\_065051153\_13749-model2.8 | -27.8068 |
|  | XP\_065051153\_13749-model2.9 | -27.6811 |
|  | XP\_065051153\_13749-model2.10 | -26.1272 |
|  | XP\_065051153\_13749-model2.11 | -25.0732 |
| # cluster 3 - Size: 13 |  |  |
|  | XP\_065051153\_13749-model3 | -30.0652 |
|  | XP\_065051153\_13749-model3.1 | -29.8463 |
|  | XP\_065051153\_13749-model3.2 | -29.5771 |
|  | XP\_065051153\_13749-model3.3 | -29.5543 |
|  | XP\_065051153\_13749-model3.4 | -28.8668 |
|  | XP\_065051153\_13749-model3.5 | -28.6921 |
|  | XP\_065051153\_13749-model3.6 | -28.5738 |
|  | XP\_065051153\_13749-model3.7 | -28.4315 |
|  | XP\_065051153\_13749-model3.8 | -28.2292 |
|  | XP\_065051153\_13749-model3.9 | -27.3895 |
|  | XP\_065051153\_13749-model3.10 | -26.6221 |
|  | XP\_065051153\_13749-model3.11 | -25.3488 |
|  | XP\_065051153\_13749-model3.12 | -23.074 |
| # cluster 4 - Size: 2 |  |  |
|  | XP\_065051153\_13749-model4 | -29.9799 |
|  | XP\_065051153\_13749-model4.1 | -27.5225 |
| # cluster 5 - Size: 2 |  |  |
|  | XP\_065051153\_13749-model5 | -29.6682 |
|  | XP\_065051153\_13749-model5.1 | -26.1278 |
| # cluster 6 - Size: 1 |  |  |
|  | XP\_065051153\_13749-model6 | -28.5863 |
| # cluster 7 - Size: 2 |  |  |
|  | XP\_065051153\_13749-model7 | -28.5281 |
|  | XP\_065051153\_13749-model7.1 | -24.9948 |
| # cluster 8 - Size: 1 |  |  |
|  | XP\_065051153\_13749-model8 | -27.1188 |
| # cluster 9 - Size: 2 |  |  |
|  | XP\_065051153\_13749-model9 | -26.6927 |
|  | XP\_065051153\_13749-model9.1 | -26.1991 |
| # cluster 10 - Size: 6 |  |  |
|  | XP\_065051153\_13749-model10 | -26.6084 |
|  | XP\_065051153\_13749-model10.1 | -26.4359 |
|  | XP\_065051153\_13749-model10.2 | -25.5762 |
|  | XP\_065051153\_13749-model10.3 | -25.34 |
|  | XP\_065051153\_13749-model10.4 | -23.1807 |
|  | XP\_065051153\_13749-model10.5 | -22.99 |
| # cluster 11 - Size: 2 |  |  |
|  | XP\_065051153\_13749-model11 | -26.0336 |
|  | XP\_065051153\_13749-model11.1 | -24.2724 |
| # cluster 12 - Size: 2 |  |  |
|  | XP\_065051153\_13749-model12 | -25.9409 |
|  | XP\_065051153\_13749-model12.1 | -15.5584 |
| # cluster 13 - Size: 1 |  |  |
|  | XP\_065051153\_13749-model13 | -23.762 |
| # cluster 14 - Size: 5 |  |  |
|  | XP\_065051153\_13749-model14 | -21.2461 |
|  | XP\_065051153\_13749-model14.1 | -20.696 |
|  | XP\_065051153\_13749-model14.2 | -19.5317 |
|  | XP\_065051153\_13749-model14.3 | -16.6478 |
|  | XP\_065051153\_13749-model14.4 | -16.597 |
| # cluster 15 - Size: 1 |  |  |
|  | XP\_065051153\_13749-model15 | -16.6141 |
| # cluster 16 - Size: 4 |  |  |
|  | XP\_065051153\_13749-model16 | -14.9051 |
|  | XP\_065051153\_13749-model16.1 | -13.6292 |
|  | XP\_065051153\_13749-model16.2 | -13.5813 |
|  | XP\_065051153\_13749-model16.3 | -12.9733 |
| # cluster 17 - Size: 4 |  |  |
|  | XP\_065051153\_13749-model17 | -14.4289 |
|  | XP\_065051153\_13749-model17.1 | -14.0415 |
|  | XP\_065051153\_13749-model17.2 | -13.9482 |
|  | XP\_065051153\_13749-model17.3 | -13.9003 |
| # cluster 18 - Size: 11 |  |  |
|  | XP\_065051153\_13749-model18 | -14.3807 |
|  | XP\_065051153\_13749-model18.1 | -14.3457 |
|  | XP\_065051153\_13749-model18.2 | -14.3329 |
|  | XP\_065051153\_13749-model18.3 | -14.3127 |
|  | XP\_065051153\_13749-model18.4 | -14.0682 |
|  | XP\_065051153\_13749-model18.5 | -14.0363 |
|  | XP\_065051153\_13749-model18.6 | -14.0348 |
|  | XP\_065051153\_13749-model18.7 | -13.9108 |
|  | XP\_065051153\_13749-model18.8 | -13.7728 |
|  | XP\_065051153\_13749-model18.9 | -13.6893 |
|  | XP\_065051153\_13749-model18.10 | -13.5251 |
| # cluster 19 - Size: 2 |  |  |
|  | XP\_065051153\_13749-model19 | -14.2234 |
|  | XP\_065051153\_13749-model19.1 | -14.0839 |
| # cluster 20 - Size: 1 |  |  |
|  | XP\_065051153\_13749-model20 | -14.1972 |
| # cluster 21 - Size: 3 |  |  |
|  | XP\_065051153\_13749-model21 | -13.8455 |
|  | XP\_065051153\_13749-model21.1 | -13.3759 |
|  | XP\_065051153\_13749-model21.2 | -13.0997 |
| # cluster 22 - Size: 1 |  |  |
|  | XP\_065051153\_13749-model22 | -13.2628 |
| # cluster 23 - Size: 1 |  |  |
|  | XP\_065051153\_13749-model23 | -12.5634 |
| # cluster 24 - Size: 2 |  |  |
|  | XP\_065051153\_13749-model24 | -11.1698 |
|  | XP\_065051153\_13749-model24.1 | -10.4153 |
| # cluster 25 - Size: 1 |  |  |
|  | XP\_065051153\_13749-model25 | -10.9689 |
| # cluster 26 - Size: 1 |  |  |
|  | XP\_065051153\_13749-model26 | -10.6965 |
| # cluster 27 - Size: 1 |  |  |
|  | XP\_065051153\_13749-model27 | -10.5482 |
| # cluster 28 - Size: 1 |  |  |
|  | XP\_065051153\_13749-model28 | -7.77076 |
|  |  |  |
